# Dietary Coenzyme Q10 Supplementation Enhances Meat Quality, Nutritional Profile, and Antioxidant Status in Meat Rabbits

**DOI:** 10.3390/ani16121807

**Published:** 2026-06-11

**Authors:** Chengfang Gao, Shikun Sun, Wenmu Zhang, Zhi Lin, Xianfeng Yan, Liya Bai, Yanru Zhang, Sican Lin, Mingming Chen, Dongjin Chen, Ming Liu, Lei Sang

**Affiliations:** 1 Fujian Key Laboratory of Animal Genetics and Breeding, Institute of Animal Husbandry and Veterinary Medicine, Fujian Academy of Agricultural Sciences, Fuzhou 350013, China; 2 Dehua County Bureau of Agricultural and Rural Affairs, Quanzhou 362500, China; 3 Longyan City Livestock Station, Longyan 364000, China; 4 Shandong Animal Husbandry Station, Jinan 250100, China; 13853181227@163.com; 5 Key Laboratory of Livestock and Poultry Multi-Omics of MARA, Institute of Animal Science and Veterinary Medicine, Shandong Academy of Agricultural Sciences, Jinan 250100, China; bailiya_2005@163.com

**Keywords:** antioxidant status, growth performance, rabbit nutrition, meat quality, fatty acid composition, intestinal morphology, endocrine regulation

## Abstract

This study evaluated the effects of dietary coenzyme Q10 (CoQ10) supplementation on growth performance, slaughter performance, meat quality, antioxidant capacity, serum profiles, and intestinal morphology in Minxinan black rabbits. A total of 250 rabbits were allocated to five dietary treatments containing 0, 30, 60, 120, or 240 mg/kg CoQ10 for 14 weeks after a 1-week adaptation period. Results indicated that supplementation with 60 mg/kg CoQ10 resulted in the highest final body weight (2.83 kg) and average daily gain (29.54 g/day), with a significantly reduced feed-to-gain ratio and mortality rate compared to the control group. Regarding slaughter performance, the 60 mg/kg group significantly reduced the abdominal fat rate. In terms of meat quality, the 60 and 120 mg/kg groups showed significantly reduced drip loss and shear force, while meat lightness (L*) increased in all supplemented groups. Cooking loss was significantly reduced in the 60 mg/kg group. Antioxidant capacity in cardiac muscle and *longissimus thoracis et lumborum* (LTL) muscle was enhanced, particularly at 60 mg/kg, with significantly elevated activities of superoxide dismutase (SOD), catalase (CAT), glutathione peroxidase (GSH-Px), and total antioxidant capacity (T-AOC), alongside reduced malondialdehyde (MDA) content. Furthermore, the 60 mg/kg group increased LTL muscle polyunsaturated fatty acid (PUFA) content, elevated serum levels of triiodothyronine (T3), growth hormone (GH), and insulin-like growth factor-1 (IGF-1), enhanced immunoglobulin concentrations, and improved intestinal morphology. In conclusion, dietary supplementation with 60 mg/kg CoQ10 improved growth performance, carcass leanness, PUFA content, and antioxidant status in broiler rabbits.

## Simple Summary

Improving production efficiency and meat quality is crucial in rabbit farming. This study investigated the effects of dietary coenzyme Q10 (CoQ10) supplementation on Minxinan black rabbits. A total of 250 rabbits were divided into five groups and fed diets containing 0 to 240 mg/kg CoQ10 for 14 weeks. The results showed that supplementation with 60 mg/kg CoQ10 had the best effects; rabbits in this group achieved the highest ADG and ADFI, with significantly reduced feed-to-gain ratio and mortality rate. Abdominal fat was reduced, and meat quality improved (lower drip loss and shear force). In addition, antioxidant capacity in the cardiac muscle and LTL muscle was enhanced. This dose also promoted PUFA content, increased levels of immunoglobulins, and villus length in the duodenum, jejunum, and ileum. In conclusion, dietary supplementation with 60 mg/kg CoQ10 improved growth performance, carcass leanness, PUFA content, and antioxidant status in broiler rabbits.

## 1. Introduction

Rabbit production has become a significant component of the global livestock industry due to its high reproductive rate, rapid growth, and providing a high-quality source of animal protein for humans [1,2]. Given the competition between crops for human consumption and animal feed, rabbits occupy a distinctive niche by efficiently converting forages and agricultural by-products into high-quality meat [3,4]. Furthermore, rabbit meat is increasingly favored by consumers for its low fat, high protein, and high biological value, positioning it as a functional food [2].

However, under modern intensive farming systems, rabbits are often exposed to various stressors, such as environmental, transport, or immune challenges. These stressors can induce excessive production of reactive oxygen species (ROS). The resulting oxidative stress impairs animal health and productivity [5,6]. This oxidative stress impairs animal growth and meat quality chiefly via muscle lipid peroxidation. This process causes deterioration in meat color, loss of flavor, and may lead to the formation of lipid oxidation products, such as malondialdehyde and 4-hydroxynonenal, which are cytotoxic aldehydes derived from lipid oxidation [7,8,9]. Additionally, oxidative stress can disrupt the intestinal epithelial integrity, compromise gut barrier function, and impair nutrient digestion and absorption, thereby posing a broader threat to overall animal health [10,11]. Therefore, enhancing the antioxidant capacity of muscle tissues in meat rabbits through nutritional regulation is an effective strategy to improve their production performance and meat quality, with the core objective of balancing high production efficiency with superior product quality and safety.

In recent years, various dietary antioxidants, including vitamins, plant extracts, and bioactive compounds, have been explored as nutritional strategies to alleviate oxidative stress and improve animal health. Among these, Coenzyme Q10 (CoQ10) has attracted increasing attention due to its dual role as both an energy metabolism regulator and a potent lipophilic antioxidant. CoQ10 is a fat-soluble quinone compound with dual biological functions. It participates in mitochondrial electron transport to promote ATP production and maintain energy supply, and concurrently acts as a crucial natural antioxidant [12]. By scavenging free radicals, CoQ10 effectively inhibits oxidative stress responses and mitigates resultant cellular damage [13]. Dietary CoQ10 supplementation has been shown to improve growth performance, antioxidant status, and meat quality in livestock [14,15,16,17].

Nevertheless, the application of CoQ10 in meat rabbit production remains insufficiently studied [18]. Most existing research has focused on the effects of CoQ10 on semen quality in heat-stressed male rabbits [14,19]; relatively few studies have examined its impact on production performance and meat quality in meat rabbits. Consequently, several critical questions persist regarding the comprehensive effects of dietary CoQ10 supplementation in rabbits. These include its potential to enhance growth performance and feed efficiency, improve carcass leanness, and modify key meat quality attributes such as water-holding capacity, tenderness, and color. In addition, its modulatory effects on serum hormone and immune profiles remain to be clarified. More specifically, further investigation is needed to determine whether CoQ10 can enhance tissue antioxidant capacity, positively influence muscle fatty acid composition, and maintain intestinal health. In the context of meat quality studies, “muscle antioxidant capacity” generally refers to skeletal muscle. Therefore, the present study assessed antioxidant capacity in both skeletal muscle and cardiac muscle. To this end, following the CoQ10 dosages used in rabbits [19] and broiler chickens [15], this study systematically evaluates the dose-dependent effects of dietary CoQ10 supplementation on a range of parameters in meat rabbits, including growth performance, slaughter performance, meat quality, muscle antioxidant capacity, muscle fatty acid profile, serum immunological and endocrine parameters, and intestinal morphology. The findings are expected to provide a scientific basis for utilizing CoQ10 as a novel, multifunctional feed additive to support the production of high-quality, nutritious, and sustainable rabbit meat.

## 2. Materials and Methods

### 2.1. Statement of Ethical Approval

The experimental protocol was reviewed and approved by the Animal Care and Use Committee of the Institute of Animal Husbandry and Veterinary Medicine, Fujian Academy of Agricultural Sciences, Fuzhou, China (Approval No. MYLISC2025-017).

### 2.2. Experimental Rabbits and Care Conditions

A total of 250 healthy weaned Minxinan Black rabbits (approximately 5 weeks old; average initial body weight 0.56 ± 0.01 kg) were used for the experiment. The experimental unit was the individual rabbit. The study was conducted under standard commercial rearing conditions without the application of any additional experimental stressors. All rabbits were kept individually in standard galvanized wire cages (40 × 60 × 45 cm) in a naturally ventilated rabbitry with free access to clean tap water. They were randomly divided into five groups of 50 rabbits each. Each rabbit was housed in a separate cage, and each cage was considered an independent replicate. Rabbits were fed twice daily, at 6:00 a.m. and 4:00 p.m., with a restricted amount of a commercial pelleted diet formulated to meet nutrition requirements [20]. The daily feed allowance was 100 g per rabbit from weaning to 60 days, and increased to 150 g per rabbit from 61 to 120 days. CoQ10 (liposoluble 99% coenzyme Q10, food grade, Huyu Biotechnology Co., Ltd., Hohhot, China) was dissolved in a small amount of edible corn oil and then mixed evenly with the basal diets to form pellet feed containing CoQ10 at concentrations of 0 (control group), 30, 60, 120, or 240 mg/kg. The composition and nutrient levels of the rabbit diet are presented in Table S1.

### 2.3. Growth Performance Analysis

Grouping was completed during the 1-week pre-trial period, with a gradual transition to the experimental diet. The formal experiment lasted 14 weeks. Initial and final body weights (FBW) were recorded at the beginning and end of this period, respectively. Daily feed intake and leftovers were weighed and recorded for each group to calculate average daily feed intake (ADFI), average daily gain (ADG), and the feed-to-gain ratio (F/G). Any incidence of mortality or signs of illness was promptly documented. The mortality rate was expressed as a percentage of the initial number of rabbits.

### 2.4. Slaughter and Meat Sampling

At the end of the experiment, eight rabbits (Rabbits with body weight close to the group mean were selected from each group, including 4 males and 4 females) per treatment group were randomly selected and slaughtered on the same day at the experimental abattoir of Dehua City Jisheng Black Rabbit Breeding Co., Ltd., Quzhou, China. After a 12 h fast, their pre-slaughter live weight was recorded. Rabbits were then humanely euthanized via cervical dislocation followed by exsanguination, and carcasses were subsequently processed in accordance with the Rabbit Slaughter Operation [21]. Carcass traits, including pre-slaughter body weight, eviscerated carcass weight, half-eviscerated carcass weight, and abdominal fat weight, were measured as described by Malgwi et al. [22]. Key metrics were calculated as follows:

- Abdominal fat rate (%) = (Abdominal fat weight/Pre-slaughter weight) × 100;
- Eviscerated yield (%)= (Eviscerated weight/Pre-slaughter weight) × 100;
- Half-eviscerated yield (%) = (Half-eviscerated weight/Pre-slaughter weight) × 100.

(Eviscerated weight excludes blood, fur, head, tail, feet, and viscera; half-eviscerated weight includes heart, liver, kidneys, and abdominal fat.)

Major organs were collected and weighed to determine organ indices (g/kg BW). All weightings were performed using a precision electronic balance (BSA224S, Sartorius, Lower Saxony, Germany) with an accuracy of 0.01 g.

### 2.5. Meat Quality Analysis

The *longissimus thoracis et lumborum* (LTL) muscles were excised from each rabbit carcass and divided into left and right subsamples. The left-side LTL muscle was assigned for sensory evaluation. Specifically, the left-side *longissimus thoracis* (LT) from approximately the 6th to the 13th thoracis vertebra was used to measure drip loss, pH values, and the left-side *longissimus lumborum* (LL) from approximately the 1st to the 6th lumbar vertebra was used to determine meat colour and cooking loss. The whole right-sided LTL muscle was allocated for chemical composition analysis.

The whole right-side LTL subsample, intended for fatty acid and antioxidant analyses, was individually packed, labeled, and transported under refrigerated conditions to the laboratory (approximately 3 h). Upon arrival, the caudal section of each LTL muscle was dissected. Visible connective tissue and external fat were trimmed, after which each sample was individually homogenized using a JB1200 benchtop food processor (Hebei Xiaojin Machinery Manufacturing Co., Ltd., Shijiazhuang, China). The homogenate was subsequently transferred into vacuum-sealed polypropylene bags and stored at −20 °C until analysis, all of which were completed within one month.

Drip loss: The left-side LT muscle from each rabbit was trimmed to a uniform size, weighed (W1), placed in a drip loss container, and stored at 4 °C for 24 h. Following storage, the sample was re-weighed (W2), and drip loss was calculated as: (W1 − W2)/W1 × 100%.

pH values: The pH value was measured 24 h post-slaughter using a portable pH meter (205, TESTO, Titisee-Neustadt, Germany) equipped with automatic temperature compensation. The pH meter was calibrated using standard buffer solutions of pH 4.01 and 6.08 prior to measurement. On the left side of the LT muscle of each rabbit, the probe was inserted to a depth of approximately 3 mm at the cranial, middle, and caudal sections. Three measurements were taken per section, and the mean pH value was recorded.

Meat colour: Colour parameters, including brightness (L*), redness (a*), and yellowness (b*), were measured using a Minolta CR-400 colorimeter (Konica Minolta Sensing, Inc., Osaka, Japan) under the CIELAB system (CIE, Comission Internationale de L’eclairage, 1976 [23]), The instrument was configured with a D65 illuminant, an 8 mm aperture, diffuse lighting, a 0° viewing angle, and the specular component included. Measurements were conducted at three distinct sites on the surface of the left-side LL muscle after allowing the meat to bloom for 30 min at 4 °C [24].

Cooking loss: The procedure followed the American Meat Science Association (1995) [25]. All samples were analyzed in a single batch. The left-side LL muscle was sliced into equal-sized pieces, boiled in water for approximately 20 min, and cooled to room temperature. Cooking loss was calculated as the percentage of weight during cooking, following the method of Chang et al. (2020) [26]: Cooking loss (%) = (W1 − W2)/W1 × 100, where W1 and W2 represent the weights of the meat sample before and after cooking, respectively. Triplicate measurements were performed for each sample.

Shear force: Shear force was determined according to Shen et al. [27]. All samples were analyzed in the same single batch for cooking loss. Cooked samples from the above procedure were cut into three uniform strips (1 × 1 × 1 cm^3^). A digital meat tenderness meter (C-LM3B, Beijing Tianxiang Feiyu Instrument and Equipment Co., Ltd., Beijing, China) was used to measure the shear force perpendicular to the muscle fibers direction. Six measurements were taken per strip, and the average value was recorded as the shear force for that sample.

Fatty acid profile: Fatty acid content was determined according to the method described by O’Fallon et al. [28]. Briefly, approximately 50 mg of LTL muscle was homogenized and mixed with 3 mL of hexane (H302-4, Thermo Fisher Scientific, Waltham, MA, USA). The mixture was shaken at 50 °C for 30 min. Subsequently, 3 mL of 0.4 mol/L methanolic potassium hydroxide solution (CAEQ-4-000306-4000, Anpel Laboratory Technologies Inc., Shanghai, China) was added, and derivatization was performed by shaking at 50 °C for an additional 30 min. After cooling to room temperature, 1 mL of water was added and thoroughly mixed. The upper organic layer was collected, and 90 µL of the supernatant was combined with 10 µL of internal standard (methyl nonadecanoate, 125 µg/mL) prior to GC-MS analysis.

Analysis was performed using a GC-MS system (8860 GC/5977C MSD, Agilent, USA) equipped with an Agilent G3903-63011 DB-FastFAME column (Agilent, Santa Clara, CA, USA). The injector temperature was set to 250 °C, with a split ratio of 10:1 and an injection volume of 1 µL. Helium was used as the carrier gas. The temperature program was as follows: initial oven temperature 50 °C (hold 0.5 min), increased at 35 °C/min to 194 °C (hold 3.5 min), then raised at 9 °C/min to 240 °C (hold 1.0 min). The interface temperature of the mass selective detector was maintained at 250 °C, with ion source and quadrupole temperatures set to 230 °C and 150 °C, respectively. Electron ionization (EI) was operated in selected ion monitoring (SIM) mode. The electron multiplier voltage mode was configured for gain factor operation, with a gain factor of 1 and an electron multiplier voltage of 1412 V. A solvent delay of 2 min was applied.

### 2.6. Determination of Antioxidant Performance Indicators

Given that CoQ10 is known to target cardiac muscle and is critical for myocardial antioxidant function, cardiac tissue was included as a predefined organ of interest in the antioxidant analysis. Cardiac muscle (left ventricular tissue) and whole right-sided LTL muscle (2–4 g each) were collected from each rabbit. Left ventricular tissue sample preparation: Visible connective tissue and external fat were trimmed from each left ventricular tissue sample. The homogenate of cardiac muscle was prepared using the same method as described above for the LTL.

The activities of superoxide dismutase (SOD), catalase (CAT), glutathione peroxidase (GSH-Px), total antioxidant capacity (T-AOC), and the level of malondialdehyde (MDA) were determined in cardiac muscle and whole right-sided LTL muscle tissues using specific commercial assay kits (Beijing Sinouk Institute of Biological Technology, Beijing, China). The optical density (OD) values were measured using a microplate reader, and the corresponding concentrations of antioxidant enzyme activities were calculated according to the formulas provided by the kit manufacturer.

### 2.7. Measurement of Serum Immune, Biochemical Indicators, and Hormone Levels

Blood samples of approximately 5 mL were collected from experimental rabbits before slaughter using specialized rabbit ear vein blood collection needles and vacuum blood collection tubes without anticoagulant. Immediately after collection, the samples were centrifuged at 4 °C (3000× *g* for 5 min) using a pre-cooled centrifuge. The serum was promptly separated and aliquoted into multiple 1.5 mL centrifuge tubes. These aliquots were transported to the laboratory on ice packs within 3 h, then frozen and stored in a freezer. When needed, one tube of serum was thawed for the detection of the following indicators. Serum immune, biochemical, and hormone indicators were analyzed following the manufacturers’ instructions. Total cholesterol (TC), triglyceride (TG), high-density lipoprotein (HDL), and low-density lipoprotein (LDL) levels were determined using kits from Zhong Sheng Bei Kong Biotechnology Co., Ltd. (China). Immunoglobulins (IgA, IgG, and IgM) and hormones, including growth hormone (GH), insulin-like growth factor-1 (IGF-1), triiodothyronine (T3), and thyroxine (T4), were quantified by enzyme-linked immunosorbent assay (ELISA) using kits purchased from Beijing Sinouk Institute of Biological Technology (China).

### 2.8. Measurement of Intestinal Morphology

Intestinal segments (approximately 1 cm in length) were immediately collected from each rabbit at sites located 3–5 cm distal to the pylorus (duodenum), 3–5 cm distal to the ligament of Treitz (jejunum), and 3–5 cm proximal to the cecum (ileum) using transverse incisions. The samples were rinsed thoroughly with physiological saline and fixed in 10% neutral buffered formalin (R21923, Thermo Fisher Scientific, USA). Paraffin embedding and sectioning were performed conventionally. Sections were observed and imaged using a slide scanner (DESK/MIDI/250/1000, 3DHISTECH Ltd., Budapest, Hungary). From each group, two representative sections were analyzed. Using Image-Pro Plus 6.0 software (calibrated at 40×), villus length, crypt depth, and mucosal thickness were measured at five locations per section (Figure S1). Three measurements were taken per section. The villus length-to-crypt depth (V/C) ratio was derived, and the mean values for each parameter were determined.

### 2.9. Statistical Analysis

The experimental data were collated in Excel 2003. Statistical analyses were performed using SPSS 26.0 (IBM Corporation, New York, NY, USA). To evaluate the dose–response relationship of CoQ10 supplementation (0, 30, 60, 120, 240 mg/kg), we conducted one-way ANOVA followed by Duncan’s test and regression-based analyses. Within the ANOVA framework, a linear polynomial contrast was applied to the ordered dose levels to test for a linear trend. Mortality rate was analyzed using the chi-square test. The following parameters were considered dependent variables: initial body weight, slaughter body weight, ADG, ADFI, F/G, carcass weight, dressing percentage, pH, L*, a*, b*, drip loss, cooking loss, shear force, organ index, fatty acid composition, antioxidant performance, serum immune and biochemical indicators, hormone levels, and intestinal morphology. In the ANOVA model, the treatment group was included as a fixed effect, and the replicate as a random effect.

To evaluate the biological consistency of treatment effects and inter-individual variability, scatter plots were employed to display antioxidant enzyme activity data from individual animals. Raw measured values from each treatment group were plotted as individual data points, overlaid with horizontal lines representing group means and error bars indicating standard deviation. Horizontal lines denote within-group means, while vertical error bars represent mean ± SE ranges, and significance letters from Duncan’s multiple comparison test (*p* < 0.05) were annotated above the upper error bar. Data points were subjected to slight random jittering along the X-axis to prevent overlapping and enhance readability.

All data are presented as mean values and standard error of the mean (SEM). A *p*-value of less than 0.05 was considered statistically significant. Bar charts, line graphs, and scatter plots were generated using GraphPad Prism 9.0 software (GraphPad Software Inc., San Diego, CA, USA), while radar charts and heat maps were created using Origin 2021 software (OriginLab Corporation, Northampton, MA, USA).

## 3. Results

### 3.1. Growth Performance and Feed Efficiency

During the 15-week experimental period, dietary CoQ10 supplementation significantly influenced growth performance (Table 1). The highest final body weight (FBW) was observed in the 60 mg/kg group, which was significantly greater than that of the control group (*p* < 0.05). A similar trend was noted for average daily gain (ADG), with the 60 mg/kg group reaching 29.54 g/day (*p* < 0.05). The feed-to-gain ratio (F/G) was significantly lower in the 60 mg/kg and 120 mg/kg groups compared with the control group (*p* < 0.05). Average daily feed intake (ADFI) remained largely unchanged across most treatment groups, except for a significant reduction in the 120 mg/kg group (*p* < 0.05). Moreover, the mortality rate decreased from 20.5% in the control group to a range of 10.4%~14.2% in the CoQ10-supplemented groups, with the 60 mg/kg, 120 mg/kg, and 240 mg/kg groups showing significantly lower rates than the control group (*p* < 0.05).

### 3.2. Slaughter Performance and Organ Development

Eviscerated yield was significantly lower in the 30 mg/kg, 60 mg/kg, and 240 mg/kg groups compared to the control and 120 mg/kg groups (*p* < 0.05). Half eviscerated yield was lowest in the 240 mg/kg group and highest in the control and 120 mg/kg groups (*p* < 0.05). Abdominal fat percentage was significantly lower in the 60 mg/kg and 120 mg/kg groups than in the control group (*p* < 0.05), indicating improved nutrient partitioning toward lean tissue deposition. Regarding organ indices, the spleen index was markedly higher in the 240 mg/kg group compared to all other groups (*p* < 0.05). The lung index was elevated in the 30 mg/kg, 60 mg/kg, and 240 mg/kg groups relative to the control and 120 mg/kg groups (*p* < 0.05). Cardiac and kidney indices showed significant differences among groups (*p* < 0.05), while the liver index also varied significantly (*p* = 0.006, Table 2).

### 3.3. Meat Quality Characteristics

**Figure 1:**
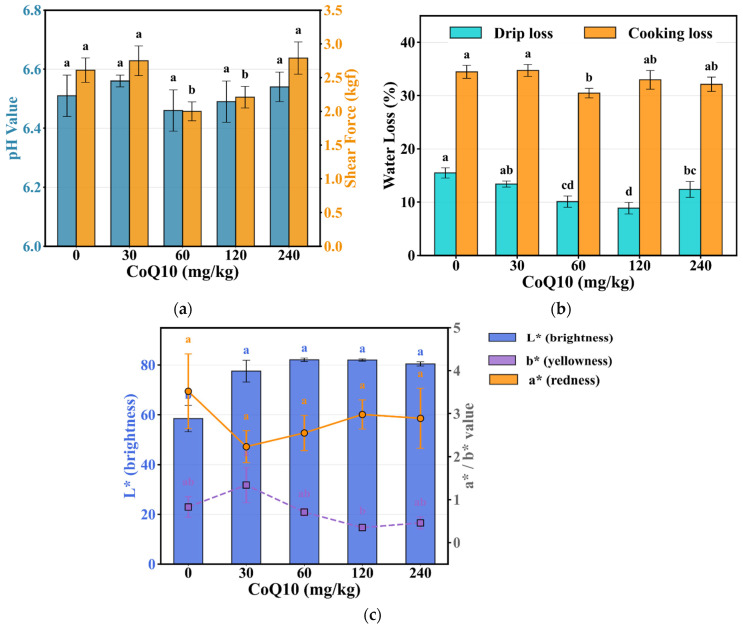
Effects of dietary CoQ10 supplementation on meat quality. (**a**) Muscle pH and moisture content; (**b**) Drip loss and cooking loss; (**c**) Meat color (L* as bar chart, left y-axis; a* and b* as line charts, right y-axis). Data are mean ± SE (*n* = 8). Different letters indicate significant differences (*p* < 0.05, Duncan’s test).

CoQ10 supplementation led to significant improvements in meat quality parameters (Figure 1a–c). The lowest drip loss was observed in the 120 mg/kg group, which was significantly lower than the control value of 8.87% (*p* < 0.05). Additionally, the 60 mg/kg group also showed a marked reduction in drip loss, reaching 10.10% compared with 15.48% in the control group (*p* < 0.05). Cooking loss was minimal in the 60 mg/kg group, reaching 30.47%, significantly lower than the control group (34.46%, *p* < 0.05, Figure 1b). Shear force indicated that tenderness in the 60 mg/kg and 120 mg/kg groups was significantly superior to the control group, with values of 2.00 kgf and 2.21 kgf, respectively, compared to 2.61 kgf in the control group (*p* < 0.05, Figure 1a). All CoQ10-supplemented groups exhibited significantly higher meat color lightness (L*) compared with the control group value of 58.46 (*p* < 0.05), with the highest value (82.08) observed in the 60 mg/kg group. Regarding redness (a*), no significant differences were detected among treatment groups (*p* > 0.05). In contrast, yellowness (b*) varied significantly, reaching its highest level in the 30 mg/kg group and its lowest level (0.35) in the 120 mg/kg group (*p* < 0.05). Furthermore, muscle pH and moisture content remained unaffected by treatment (*p* > 0.05, Figure 1c).

### 3.4. Effects of CoQ10 on Muscle Fatty Acid Composition

**Figure 2:**
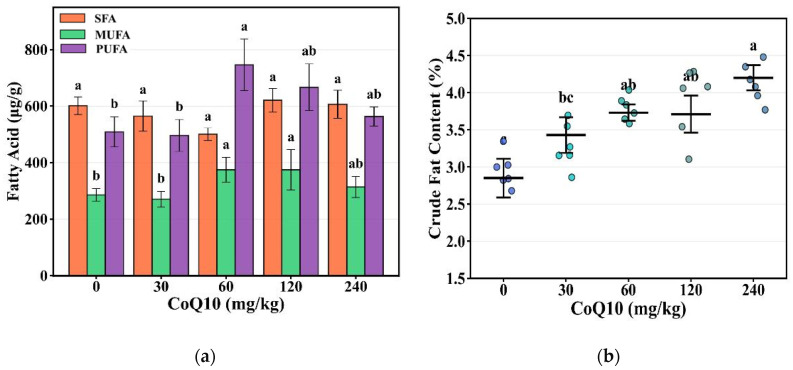
Effects of dietary CoQ10supplementation on muscle fatty acid composition. Data are mean ± SE (*n* = 8). Different letters indicate significant differences (*p* < 0.05, Duncan’s text).

As illustrated in Figure 2, CoQ10 supplementation exerted significant effects on muscle lipid composition. Crude Fat content increased with supplementation level, reaching 4.2% in the 240 mg/kg group compared to 2.85% in the control group (*p* < 0.05, Figure 2b).

Total polyunsaturated fatty acid (PUFA) content was highest in the 60 mg/kg group, reaching 745.71 μg/g, significantly higher than the control group (509.15 μg/g, *p* < 0.05). Monounsaturated fatty acids (MUFA) in both the 60 mg/kg and 120 mg/kg groups were significantly higher than the control group, reaching 375.07 μg/g and 374.25 μg/g, respectively, compared to 285.89 μg/g in the control group (*p* < 0.05), while saturated fatty acid (SFA) content remained stable (500.72–620.74 μg/g, *p* > 0.05, Figure 2a).

C18:3n3 in the 120 mg/kg and 240 mg/kg groups was significantly higher than the control group (*p* < 0.05), and it belongs to the ω-3 PUFA category. C18:2n6c belongs to the ω-6 PUFA category, and it was also increased in the 60 mg/kg group, reaching 551.96 μg/g, higher than the control group (*p* < 0.05). Among individual fatty acids, palmitoleic acid (C16:1) content was substantially higher in the 60 mg/kg and 120 mg/kg groups, reaching 14.59 μg/g and 15.62 μg/g, respectively, compared to 3.51 μg/g in the control group (*p* < 0.05, Table 3).

### 3.5. Serum Immunological and Metabolic Parameters

**Figure 3:**
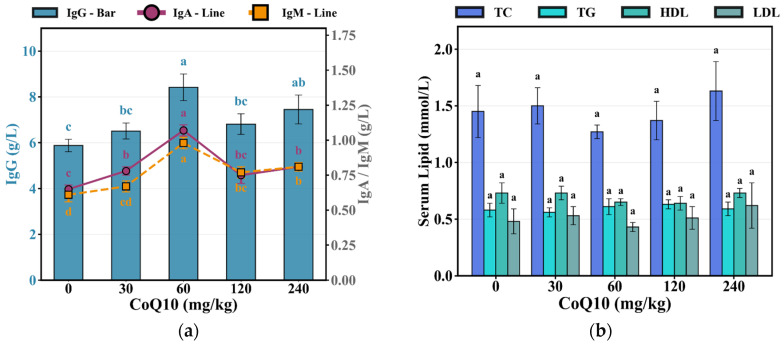
Effects of dietary CoQ10 supplementation on serum immunological and metabolic parameters. (**a**) Immunoglobulins (IgG as bar chart, left y-axis; IgA and IgM as line charts, right y-axis); (**b**) Lipid profiles (TC, TG, HDL, LDL). Data are mean ± SE (*n* = 8). Different letters indicate significant differences (*p* < 0.05, Duncan’s test).

Serum immunoglobulin concentrations increased significantly with CoQ10 supplementation (Figure 3a,b). IgG reached 8.42 g/L in the 60 mg/kg group compared to 5.88 g/L in the control group (*p* < 0.05), with the 240 mg/kg group also exhibiting elevated levels of 7.45 g/L (*p* < 0.05). IgA was highest in the 60 mg/kg group, reaching 1.07 g/L, higher than the control group (0.65 g/L, *p* < 0.05), indicating enhanced mucosal immunity. Similarly, IgM increased to 0.98 g/L in the 60 mg/kg group compared to 0.61 g/L in the control group (*p* < 0.05). IgA, IgG and IgM concentrations in the 60 and 240 mg/kg groups were significantly higher than those in the control group (*p* < 0.05), indicating effective activation of humoral immune responses (Figure 3a). Serum lipid profiles, including total cholesterol (1.27–1.63 mmol/L), triglycerides (0.56–0.63 mmol/L), high-density lipoprotein (0.64–0.73 mmol/L), and low-density lipoprotein (0.43–0.62 mmol/L), were unaffected by CoQ10 supplementation (*p* > 0.05, Figure 3b), maintaining normal physiological ranges across all treatment groups.

### 3.6. Tissue Antioxidant Defense Systems

**Figure 4:**
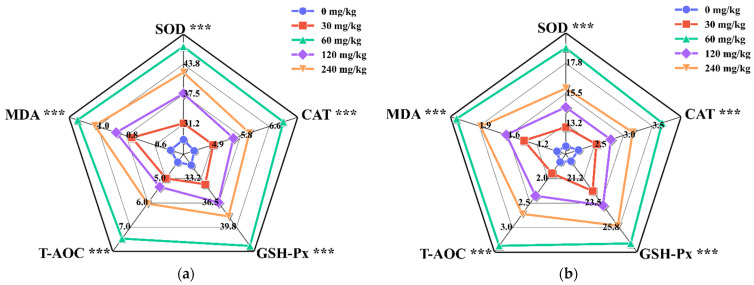
Antioxidant capacity in rabbit tissues (radar charts). (**a**) cardiac muscle; (**b**) LTL muscle. Five parameters shown: SOD, CAT, GSH-Px, T-AOC, and MDA. Each axis represents actual value range with normalized data points. MDA is inversely normalized. Different colors/markers represent CoQ10 levels (0–240 mg/kg). Data are mean ± SE (*n* = 8). *** indicates *p* < 0.01.

CoQ10 supplementation significantly enhanced antioxidant enzyme activities in both cardiac muscle and LTL muscle (Figure 4a,b). Comprehensive radar plot analysis demonstrated coordinated upregulation of all five antioxidant parameters in both tissue types, with the 60 mg/kg group showing the most pronounced improvements across all parameters (*p* < 0.05). In cardiac muscle (Figure 4a), SOD was highest in the 60 mg/kg group, reaching 47.43 U/mg compared to 28.07 U/mg in the control group (*p* < 0.05). CAT increased from 4.33 U/mg in the control group to 7.06 U/mg in the 60 mg/kg group (*p* < 0.05), facilitating hydrogen peroxide detoxification. GSH-Px reached 42.25 U/mg in the 60 mg/kg group compared to 31.45 U/mg in the control group (*p* < 0.05), while T-AOC increased from 4.32 to 7.47 U/mg (*p* < 0.05). MDA content, a marker of lipid peroxidation, was substantially reduced in the 60 mg/kg group, reaching 0.46 nmol/mg compared to 1.11 nmol/mg in the control group (*p* < 0.05). The 120 mg/kg and 240 mg/kg groups also exhibited significant antioxidant improvements, although generally at intermediate levels between the 60 mg/kg group and the control group. Following CoQ10 supplementation, antioxidant capacity in the LTL muscle was significantly enhanced (Figure 4b). SOD activity in the 60 mg/kg group reached 18.89 U/mg, significantly higher than the control group (11.59 U/mg, *p* < 0.05). CAT increased from 2.22 to 3.67 U/mg (*p* < 0.05), GSH-Px increased from 19.64 to 27.20 U/mg (*p* < 0.05), and T-AOC increased from 1.66 to 3.37 U/mg (*p* < 0.05). MDA content decreased from 2.10 nmol/mg in the control group to 0.97 nmol/mg in the 60 mg/kg group (*p* < 0.05). The levels of all antioxidant parameters in both tissue types were significantly altered depending on the dosage of CoQ10 supplementation.

### 3.7. Endocrine Regulation and Intestinal Architecture

**Figure 5:**
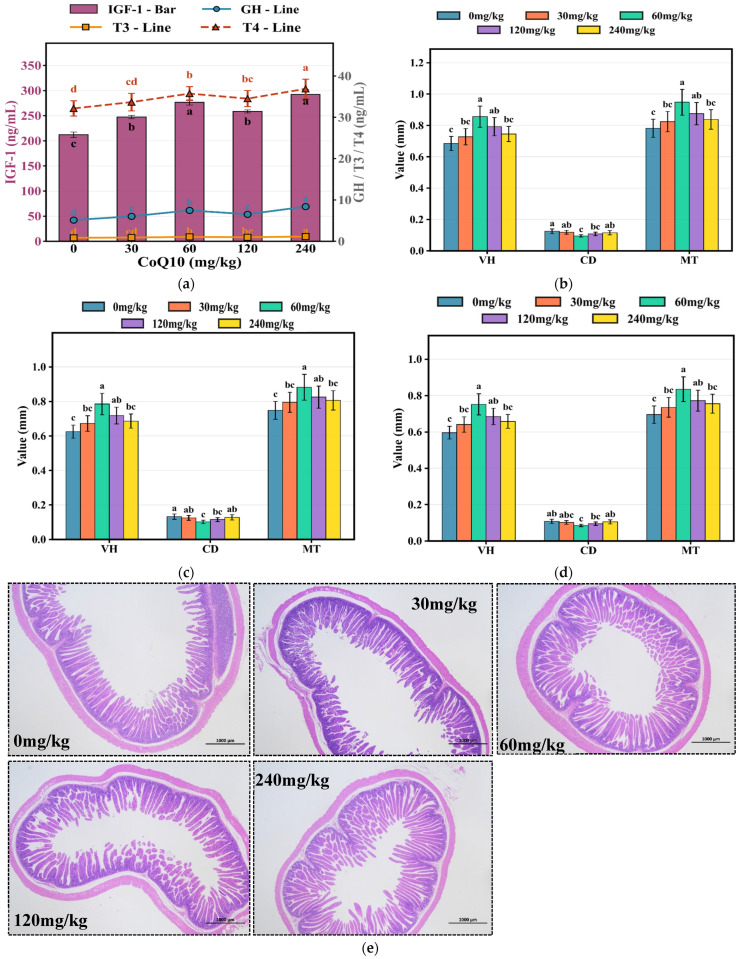
Hormones and intestinal morphology. (**a**) Serum hormones (IGF-1 bar, GH/T3/T4 lines); (**b**) Duodenum; (**c**) Jejunum; (**d**) Ileum (VL, CD, MT); (**e**) Ileal tissue sections (HE, 40×). Data are mean ± SE (*n* = 8). Different letters indicate *p* < 0.05 (Duncan’s test). VL: villus length, CD: crypt depth, MT: mucosal thickness.

Serum hormones were significantly modulated by CoQ10 supplementation (Figure 5a). GH concentration was highest in the 240 mg/kg group, reaching 8.39 ng/mL, followed by the 60 mg/kg group (7.48 ng/mL), both significantly higher than those of the control group (5.14 ng/mL, *p* < 0.05). IGF-1 exhibited its peak concentrations in the 240 mg/kg and 60 mg/kg groups, reaching 292.29 ng/mL and 276.54 ng/mL, respectively, significantly exceeding the control group (212.19 ng/mL, *p* < 0.05). T3 levels progressively increased, reaching maximum concentrations in the 240 mg/kg and 60 mg/kg groups at 1.15 ng/mL and 1.06 ng/mL, respectively, both higher than those in the control group (0.85 ng/mL, *p* < 0.05). The T4/T3 ratio was maintained within normal physiological ranges (30-40:1) across all treatment groups, indicating coordinated thyroid axis activation without disrupting hormone conversion. Intestinal morphometric analysis revealed substantial structural improvements following CoQ10 supplementation. In the duodenum, the 60 mg/kg group exhibited increased villus length (0.856 mm), reduced crypt depth (0.095 mm), and mucosal thickness (0.948 mm), compared to 0.685 mm, 0.125 mm, and 0.782 mm, respectively, in the control group (*p* < 0.05, Figure 5b). The jejunum demonstrated significant improvements in the 60 mg/kg group, with villus length reaching 0.785 mm, crypt depth of 0.102 mm, and mucosal thickness of 0.882 mm, compared to corresponding values of 0.625 mm, 0.132 mm, and 0.748 mm in the control group (*p* < 0.05, Figure 5c). Similarly, ileum measurements in the 60 mg/kg group showed villus length of 0.752 mm, crypt depth of 0.085 mm, and mucosal thickness of 0.835 mm, compared to corresponding values of 0.596 mm, 0.108 mm, and 0.695 mm in the control group (*p* < 0.05), Furthermore, the 120 mg/kg and 240 mg/kg groups also exhibited significant improvements relative to the control group (*p* < 0.05, Figure 5d). As illustrated in Figure 5e, the CoQ10-supplemented groups on the ileum’s effects, particularly the 60 mg/kg group, exhibited more clearly defined villus structures, intact epithelial integrity, and robust mucosal architecture, contrasting with the shorter, less organized villi observed in control animals (Figure 5e).

### 3.8. Dose–Response Relationships of Antioxidant Indices

**Figure 6:**
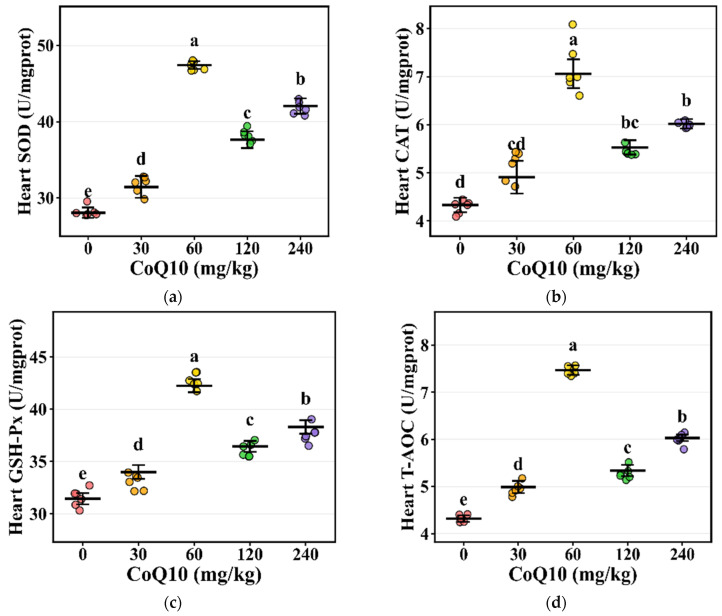

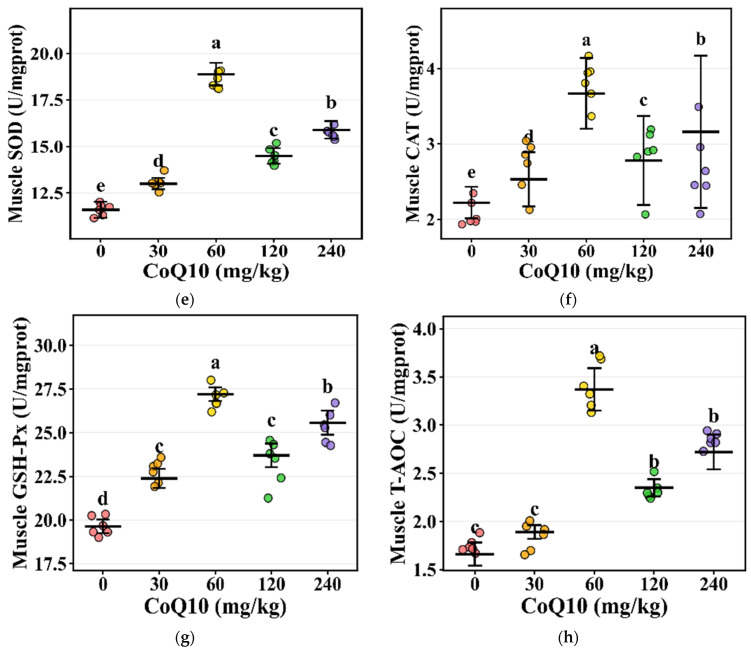
Individual antioxidant enzyme data. (**a**–**d**) Cardiac muscle: SOD, CAT, GSH-Px, T-AOC; (**e**–**h**) LTL muscle: SOD, CAT, GSH-Px, T-AOC. Individual points (*n* = 8), mean, and SE shown. Colors represent CoQ10 levels. Different letters indicate *p* < 0.05 (Duncan’s test).

Scatter plot analysis revealed clear relationships between CoQ10 supplementation dose and antioxidant indices (Figure 6a–h). For cardiac tissue, the distribution of individual data points for SOD (Figure 6a), CAT (Figure 6b), GSH-Px (Figure 6c), and T-AOC (Figure 6d) across dose groups demonstrated distinct dose-dependent trends, with enzyme activity values in the 60 mg/kg group significantly higher than those in the other dose groups. Notably, individual data points within the 60 mg/kg group clustered tightly, with smaller coefficients of variation within the group, whereas the control group and high-dose group (240 mg/kg) exhibited greater inter-individual fluctuations. Similar patterns were observed in skeletal muscle tissue, where scatter plot distributions for SOD (Figure 6e), CAT (Figure 6f), GSH-Px (Figure 6g), and T-AOC (Figure 6h) all demonstrated that the 60 mg/kg group showed optimal enzyme activity levels and minimal within-group variation. The orderly distribution of individual data points across dose groups confirmed that antioxidant improvements were not driven by individual outliers but rather represented genuine and reproducible population-level biological responses, providing robust experimental evidence for establishing 60 mg/kg as the optimal supplementation dose.

## 4. Discussion

### 4.1. CoQ10 Promotes Growth Performance via GH/IGF-1 Axis and Intestinal Morphology

The present study found that in the 60 mg/kg CoQ10 group, the average daily feed intake of rabbits remained unchanged, while the average daily gain significantly increased, leading to improved feed efficiency. Serum GH and IGF-1 levels in this group were also higher than those in other groups. GH is secreted by the pituitary gland and acts on the liver to stimulate IGF-1 production. Accordingly, these observations suggest that, although direct evidence of signaling pathway activation was not examined in this study, CoQ10 may protect the endocrine function of the pituitary-liver axis and alleviate suppression of the GH/IGF-1 axis, thereby improving growth performance at the endocrine level. Previous studies have shown that hepatic IGF-1 synthesis is an energy-demanding process sensitive to the body’s energy status, and CoQ10 supplementation may stimulate the GH/IGF-1 axis by optimizing hepatocellular energy metabolism [17,29,30]. This finding is consistent with reports by Galetaki et al. and Zargari et al. [31,32], both demonstrating that CoQ10 supplementation can preserve endocrine function, maintain metabolic homeostasis, and enhance stress resistance under conditions such as neonatal hypoxia or ammonia stress by restoring or upregulating IGF-1 and insulin levels. Furthermore, endocrine improvement can directly translate into enhanced growth performance [17,29], as activation of the GH/IGF-1 axis may enhance protein synthesis, inhibit protein degradation, enhance skeletal muscle development, and improve feed utilization [33,34,35,36]. Thus, the superior growth performance observed at the 60 mg/kg CoQ10 group likely reflects the external manifestation of this endocrine regulatory effect. The absence of additional growth promotion at higher CoQ10 doses (120 and 240 mg/kg) suggests a plateau or mild feedback inhibition of the GH/IGF-1 axis, possibly due to negative endocrine feedback or metabolic saturation, indicating a nonlinear pattern of growth response to CoQ10 supplementation. In addition, activation of the GH/IGF-1 axis significantly affects thyroid hormone metabolism. Hussain et al. (1996) [37] demonstrated that both GH and IGF-1 can increase the conversion efficiency of T4 to T3. In the present study, the T4/T3 ratio remained within the normal range (30-40:1). This observation, together with GH/IGF-1 axis activation, collectively suggests that CoQ10 promotes positive endocrine interactions. Furthermore, the present study found that at 60 mg/kg CoQ10, rabbit intestinal villus height increased, accompanied by a decrease in crypt depth. Figure 5e clearly shows intact villus structure and a continuous epithelial cell layer in the 60 mg/kg group. The improvement in intestinal morphology, characterized by increased villus height and decreased crypt depth, expands the intestinal absorptive surface area and reduces cell turnover rate, thereby enhancing digestive and absorptive capacity, reducing energy expenditure for intestinal maintenance, and ultimately improving feed efficiency. This finding aligns with studies by Liu et al. and Shimizu et al. [38,39], which consistently showed that dietary CoQ10 supplementation ameliorates radiation-induced enteropathy and enhances growth performance and stress resistance in Litopenaeus vannamei by protecting intestinal structure and enhancing antioxidant capacity. Increased villus height expands the absorptive surface area [40], while decreased crypt depth indicates a reduced cell turnover rate [41]. These changes collectively reduce the energy expenditure required for intestinal maintenance, as intestinal epithelial cells are among the most metabolically active cells in the body, undergoing complete renewal every 3–5 days in rabbits [42,43].

### 4.2. Effects of CoQ10 on Meat Quality

The significant reductions in drip loss and shear force can be attributed to antioxidant mechanisms that protect muscle tissue integrity [11]. However, changes in intramuscular fat content and lipid composition may also influence water retention and muscle physicochemical properties, an aspect that warrants integrated consideration [44,45]. Water-holding capacity is primarily determined by the integrity of myofibrillar proteins and the sarcolemma [44] but also by the hydrophobic interactions mediated by intramuscular lipids [44]. These structural components are particularly susceptible to oxidative damage during pre-slaughter stress and post-mortem aging. Reduced muscle malondialdehyde content indicates suppression of lipid peroxidation, protecting phospholipid bilayers and protein-water binding capacity [21]. In parallel, the moderate increase in intramuscular fat observed at 240 mg/kg CoQ10 may contribute to improved water retention by physically stabilizing the muscle matrix and reducing exudate channels. Oxidative stress-induced protein carbonylation and disulfide bond formation disrupt muscle structure, leading to decreased water-holding capacity [1]. Tenderness improvement involves multiple mechanisms. Elevated antioxidant enzyme activities (Figure 6e–h) help protect calpains and cathepsins from oxidative inactivation, facilitating appropriate post-mortem proteolysis [46]. Moderate increases in intramuscular fat further improve tenderness through physical separation of muscle fiber bundles [47]. In the present study, the relatively modest increase in intramuscular fat, compared to the pronounced changes in antioxidant enzyme activities, suggests that antioxidant-mediated protection of myofibrillar proteins may be a key factor contributing to the improved tenderness. Nevertheless, the concurrent elevation of PUFAs and total lipids may synergistically enhance tenderness by altering membrane fluidity and reducing cross-bridge formation between myofilaments [48]. The tight clustering of individual antioxidant enzyme data points at 60 mg/kg CoQ10 (Figure 6) indicates stable and reliable treatment effects with low inter-individual variation. Stable muscle pH (6.46–6.56) demonstrates that CoQ10 did not interfere with glycolysis, thereby supporting the maintenance of processing suitability for meat products. In addition, significant improvements in meat color lightness have important commercial implications, as meat color is one of the primary determinants of consumer purchasing decisions [6]. Elevated L* values indicate reduced myoglobin oxidation, with oxymyoglobin maintaining its bright red color rather than oxidizing to brown metmyoglobin [49]. This color stability enhances consumer acceptability [2].

### 4.3. Effects of CoQ10 on PUFA Content

The present study found that after dietary CoQ10 supplementation, the antioxidant capacity of rabbit meat and the total PUFA content increased simultaneously (from 509.15 to 745.71 μg/g). This is explained by the dual biological functions of CoQ10. As a lipophilic antioxidant embedded in biological membranes, CoQ10 effectively scavenges free radicals in the lipid peroxidation chain reaction, thereby interrupting the auto-oxidation process of PUFAs [50,51]. In the absence of exogenous antioxidant supplementation, endogenous PUFAs in muscle tissue are highly susceptible to oxidative attack and depletion, leading to decreased content [51,52]. However, after CoQ10 supplementation, these oxidizable PUFAs are protected and thus retained or even enriched in tissues [53]. Observed increase in PUFA content is not due to CoQ10 promoting PUFA synthesis or deposition, but rather because the portion that would otherwise be lost to oxidation is preserved. A similar phenomenon has been confirmed in a rabbit atherosclerosis model, where CoQ10 supplementation significantly reduced aortic lipid peroxide levels while tissue lipid composition with respect to PUFAs did not undergo unfavorable changes, suggesting that CoQ10 primarily plays a protective rather than a metabolic regulatory role [54]. Furthermore, in rabbit meat quality studies, when diets rich in PUFA-containing flaxseed were supplemented with antioxidants, muscle ω-3 PUFA content significantly increased along with enhanced oxidative stability, and the researchers similarly attributed this to the enhancement of the endogenous antioxidant defense system [55]. In the present study, ω-6 and ω-3, as the main components of PUFAs, exhibited a concurrent increasing trend. Although the ω-6/ω-3 ratio (37:1) remained higher than the ideal target of 5:1 from a human nutritional perspective [56,57], the substantial increase in ω-3 PUFA content makes CoQ10-supplemented rabbit meat a viable terrestrial source of ω-3 products. This phenomenon has not been reported in previous studies and represents a novel finding of the present research, which is of particular importance for populations with fish allergies or limited access to marine resources. Thus, CoQ10 protects PUFAs from oxidative loss, achieving effective retention while improving meat oxidative stability.

### 4.4. Effects of CoQ10 on Antioxidant Capacity

Santos et al. [58] found that the coordinated elevation of SOD, CAT, and GSH-Px activities in cardiac and skeletal muscle reflects a systemic enhancement of the body’s oxidative defense capacity. The present study further confirmed that at 60 mg/kg CoQ10, the activities of antioxidant enzymes in both cardiac and skeletal muscle of rabbits were significantly increased. Scatter plot analysis (Figure 6a–h) further showed that inter-individual variation within this group was minimal, in contrast to the greater variability observed in the 0 and 240 mg/kg CoQ10 groups. This consistency at the population level is of great significance for the reliable application of CoQ10 in commercial rabbit production. Mechanistically, CoQ10 may exert its effects through direct free radical scavenging and potentially through the Nrf2-ARE pathway [59]. However, this study did not directly measure Nrf2 nuclear translocation or the expression of downstream targets such as HO-1 and NQO1. Therefore, this interpretation remains speculative and requires confirmation in future mechanistic studies. The more pronounced effects observed in cardiac muscle compared to skeletal muscle can be attributed to its higher mitochondrial density and reactive oxygen species (ROS) production rate [60]. The cardioprotective effects observed in this study are consistent with clinical evidence indicating that CoQ10 supplementation reduces cardiovascular events in heart failure patients [61,62], supporting the translational value of this research. The positive role of CoQ10 in enhancing systemic antioxidant defense and increasing antioxidant enzyme levels has been confirmed in various animal species, including humans [62,63], mice [64], and dairy cows [17]. Similar dose-dependent antioxidant responses have been reported in broiler chickens supplemented with CoQ10, where intermediate doses produced more consistent effects than higher doses [15].

### 4.5. Practical Implications and Dose Optimization of CoQ10

Growth, meat quality, and antioxidant parameters peaked at 60 mg/kg CoQ10, whereas GH, IGF-1, and T4 levels continued to rise at higher doses. This dissociation suggests that while endocrine responses may further increase with higher doses, the functional outcomes for growth and meat quality reach an optimum at 60 mg/kg. The diminished efficacy at doses above 60 mg/kg may be attributed to the saturation of CoQ10 absorption and tissue uptake. At supraoptimal concentrations, excess CoQ10 may undergo incomplete reduction in the mitochondrial electron transport chain, leading to semiquinone radical accumulation and paradoxical ROS generation [65]. This unexpected increase in ROS can subsequently trigger oxidative damage to mitochondrial lipids, proteins, and mtDNA, potentially compromising cellular energy metabolism and activating apoptotic pathways. Therefore, careful titration of CoQ10 dosage is critical when using it as a dietary supplement or therapeutic agent. Additionally, excessive lipophilic compounds may interfere with the absorption of other fat-soluble vitamins through competitive mechanisms at the intestinal level [66].

Commercial applications can adjust dosing based on objectives. When prioritizing growth rate and fatty acid composition, 60 mg/kg is optimal. When emphasizing endocrine or immune function, 120 and 240 mg/kg CoQ10 may be considered, though economic viability requires evaluation. Rabbit meat enriched with CoQ10 provides a source of ω-3, effectively meeting contemporary nutritional challenges. Current diets contain excessive ω-6 and insufficient ω-3, promoting inflammation and cardiovascular disease [57]. While fish represent the primary ω-3 source, they face sustainability, contamination, and allergy concerns. Rabbit meat is inherently lean and high in protein. When enriched with CoQ10, it can serve as a terrestrial alternative and offer both high-quality animal protein and a bioavailable source of CoQ10 without the environmental concerns associated with overfishing. The rabbit model exhibits lipoprotein metabolism and atherosclerosis susceptibility similar to humans [67]. The cardioprotective effects, lipid profile optimization, and hormonal regulation observed in this rabbit study may offer mechanistic insights into the cardiovascular benefits of CoQ10 reported in humans. These insights are consistent with large-scale studies associating CoQ10 with reduced cardiovascular mortality [68] and with clinical trials showing benefits in patients with heart failure [61].

### 4.6. Immune Enhancement and Metabolic Safety

Significant elevations in IgG, IgA, and IgM indicate enhanced humoral immunity [69]. Immunoglobulin synthesis is an energy-intensive process, and the enhanced systemic antioxidant capacity observed in the present study (increased SOD, CAT, and GSH-Px activities) may protect activated lymphocytes from oxidative damage during the immune response, thereby supporting antibody production [70]. Furthermore, maintenance of normal serum lipid profiles is particularly important, as certain growth promoters (e.g., ractopamine, beta-adrenergic agonists) may elevate blood lipids and increase cardiovascular risk [71]. In the present study, TC, TG, HDL, and LDL all remained within normal ranges, indicating that growth enhancement was achieved without metabolic disruption or lipid accumulation.

### 4.7. Limitations and Future Directions

Several limitations of this study should be acknowledged. Within the mitochondrial electron transport chain, CoQ10 (ubiquinone) accepts electrons from complexes I and II and transfers them to complex III, a crucial step for proton gradient formation and ATP synthesis [72]. In its reduced form (ubiquinol), CoQ10 also functions as a lipid-soluble antioxidant that directly scavenges reactive oxygen species generated during oxidative phosphorylation, thereby protecting mitochondrial membranes, proteins, and DNA from oxidative damage [73]. The present study has confirmed its antioxidant function. However, whether this function is dependent on mitochondrial electron transport remains to be elucidated. Therefore, future mechanistic studies are needed to clarify the synergistic relationship between the mitochondrial electron transport function and the antioxidant function of CoQ10, thereby providing a more robust theoretical foundation for its pharmaceutical development. Furthermore, although the present study found that CoQ10 has a positive effect on the enrichment of PUFAs in fresh rabbit meat, further investigation is required into the storage stability of PUFA-enriched rabbit meat at the biochemical level, including its resistance to lipid oxidation during refrigerated and frozen storage.

## 5. Conclusions

In summary, this study demonstrates that dietary CoQ10 supplementation, particularly at an optimal dose, significantly improves growth performance, meat quality, and the fatty acid composition in meat rabbits. 60 mg/kg CoQ10 treatment effectively increased final body weight, reduced feed conversion ratio, and enhanced multiple meat quality traits, including drip loss, shear force, and color lightness. Notably, it elevated muscle ω-3 polyunsaturated fatty acid content, positioning CoQ10-supplemented rabbit meat as a potential viable terrestrial source of cardioprotective fatty acids.

## Figures and Tables

**Table 1:** Effects of diets supplemented with CoQ10 on growth performance in Minxinan black rabbits.

Items	CoQ10 Levels (mg/kg Diet), *n* = 50					SEM ^1^	*p*-Value		
	0	30	60	120	240		*G*	*L*	*Q*
Initial body weight (IBW, kg)	0.54	0.57	0.58	0.56	0.55	0.01	/	/	/
Final Body weight (FBW, kg)	2.54 ^c^	2.69 ^b^	2.83 ^a^	2.67 ^b^	2.61 ^bc^	0.03	0.001	0.959	0.003
Mortality rate (%)	20.50 ^a^	14.20 ^ab^	12.30 ^b^	10.40 ^b^	12.50 ^b^	0.12	/	/	/
Average daily gain (ADG, g)	26.43 ^b^	26.75 ^b^	29.54 ^a^	27.39 ^ab^	27.85 ^b^	0.97	0.001	0.168	0.027
Average daily feed intake (ADFI, g)	145.84 ^ab^	144.33 ^ab^	144.27 ^ab^	134.00 ^b^	150.65 ^a^	3.45	0.297	0.279	0.087
Feed-to-gain ratio (F/G)	5.38 ^a^	5.31 ^a^	4.79 ^b^	4.76 ^b^	5.46 ^a^	0.20	0.076	0.705	0.008

^1^ SEM indicates standard error of mean (*n* = 50). *G*, *L*, and *Q* represent the *p*-values for group, linear, and quadratic effects, respectively. Different letters indicate significant differences, whereas the same letter, or the absence of a letter, indicates no significant difference (*p* < 0.05, Duncan's test and regression-based analyses).

**Table 2:** Effects of dietary CoQ10 supplementation on carcass traits and organ indices.

Items	CoQ10 Levels (mg/kg Diet), *n* = 8					SEM ^1^	*p*-Value		
	0	30	60	120	240		*G*	*L*	*Q*
Eviscerated yield (%)	51.21 ^a^	49.89 ^c^	50.07 ^bc^	50.63 ^ab^	48.46 ^d^	1.18	<0.001	<0.001	0.195
Half eviscerated yield (%)	54.62 ^a^	53.21 ^b^	53.45 ^ab^	54.54 ^a^	52.31 ^c^	1.25	<0.001	<0.001	0.006
Abdominal fat rate (%)	2.80 ^a^	2.77 ^a^	2.09 ^c^	2.48 ^b^	2.56 ^ab^	0.34	<0.001	0.181	<0.001
Cardiac index (g·kg^−1^)	2.7 ^a^	2.72 ^a^	2.51 ^b^	2.52 ^b^	2.50 ^b^	0.16	0.001	0.001	0.027
Liver index (g·kg^−1^)	68.20 ^ab^	64.63 ^bc^	63.01 ^c^	69.18 ^a^	63.41 ^c^	4.40	0.006	0.215	0.465
Spleen index (g·kg^−1^)	0.41 ^b^	0.396 ^b^	0.45 ^b^	0.45 ^b^	0.65 ^a^	0.19	<0.001	<0.001	0.048
Lung index (g·kg^−1^)	4.85 ^b^	5.22 ^a^	5.23 ^a^	4.53 ^b^	5.25 ^a^	0.44	<0.001	0.404	0.072
Kidney index (g·kg^−1^)	5.47 ^b^	5.32 ^bc^	5.46 ^bc^	5.24 ^c^	5.64 ^a^	0.18	0.002	0.019	0.001

^1^ Data are mean ± SE (*n* = 8). Different letters indicate significant differences, whereas the same letter, or the absence of a letter, indicates no significant difference (*p* < 0.05, Duncan's test and regression-based analyses). G, L, and Q represent the *p*-values for group, linear, and quadratic effects, respectively.

**Table 3:** Effect of diets supplemented with CoQ10 on the content of fatty acids in meat (μg/g).

Items	CoQ10 Levels (mg/kg Diet), *n* = 8					SEM ^1^	*p*-Value ^2^
	0	30	60	120	240		
C6:0	0.08 ^b^	0.13 ^b^	0.23 ^a^	0.25 ^a^	0.23 ^a^	0.01	<0.001
C10:0	0.11 ^ab^	0.06 b	0.11 ^ab^	0.18 ^a^	0.07 ^b^	0.03	<0.001
C12:0	0.73	0.52	0.70	0.75	0.52	0.15	0.216
C13:0	0.12	0.12	0.10	0.14	0.10	0.02	0.145
C14:0	12.65	12.52	9.41	13.24	9.72	3.16	0.387
C16:0	446.78 ^a^	391.11 ^b^	389.32 ^b^	475.70 ^a^	406.19 ^b^	40.56	0.006
C16:1	3.51 ^b^	8.55 ^ab^	14.59 ^a^	15.62 ^a^	8.56 ^ab^	2.59	0.016
C18:1 n9c	269.23 ^b^	285.39 ^b^	335.02 ^a^	314.53 ^a^	246.32 ^b^	21.65	0.035
C18:3 n3	5.59 ^b^	6.37 ^b^	7.17 ^b^	10.06 ^a^	8.33 ^a^	1.60	0.510
C18:2 n6c	322.25 ^b^	312.30 ^b^	551.96 ^a^	477.54 ^ab^	365.06 ^b^	50.62	0.033
C20:1	3.46	4.14	3.55	4.02	3.41	1.36	0.513
C20:2	5.26	5.84	5.98	5.75	5.68	0.54	0.940
C20:3 n6	7.75 ^b^	8.84 ^a^	8.23 ^a^	9.18 ^a^	8.38 ^a^	1.02	0.041
C20:4 n6	130.93 ^b^	129.66 ^b^	140.86 ^a^	142.39 ^a^	138.82 ^ab^	11.35	0.048
C20:5n3	2.71	2.35	2.22	2.02	2.51	0.25	0.851

^1^ SEM indicates standard error of mean. ^2^ *p*-values indicate the significance of differences between groups. Different letters denote statistical significance (*p* < 0.05), while the absence of letters indicates no significant difference (*p* ≥ 0.05).

## Data Availability

Data will be made available on request.

## Supplementary Materials

The following supporting information can be downloaded at https://www.mdpi.com/article/10.3390/ani16121807/s1: Figure S1. Representative image showing morphology measurement in intestinal tissue. Crypt depth (yellow arrows), Villus length (red arrows), and Mucosal thickness (blue arrows) are indicated; Table S1. Composition and nutrient levels of the commercial pellet diet (air-dry basis, %).

## Abbreviations

The following abbreviations are used in this manuscript:

CoQ10	Coenzyme q10
LTL	Longissimus thoracis et lumborum
SOD	Superoxide dismutase
CAT	Catalase
GSH-Px	Glutathione peroxidase
T-AOC	Total antioxidant capacity
MDA	Malondialdehyde
PUFA	Polyunsaturated fatty acid
T3	Triiodothyronine
GH	Growth hormone
IGF-1	Insulin-like growth factor-1
ROS	Reactive oxygen species
FBW	Final body weight
ADFI	Average daily feed intake
ADG	Average daily gain
F/G	Feed-to-gain ratio
*L**	Lightness
*a**	Redness
*b**	Yellowness
LT	Longissimus thoracis
LL	Longissimus lumborum
TC	Total cholesterol
TG	Triglyceride
HDL	High-density lipoprotein
LDL	Low-density lipoprotein
IgA	Immunoglobulin a
IgG	Immunoglobulin g
IgM	Immunoglobulin m
T4	Thyroxine
V/C	Villus length-to-crypt depth ratio
SFA	Saturated fatty acid
MUFA	Monounsaturated fatty acid
ARE	Antioxidant response element
Nrf2	Nuclear factor erythroid 2-related factor 2
