# Supplementary material for: Dietary Coenzyme Q10 Supplementation Enhances Meat Quality, Nutritional Profile, and Antioxidant Status in Meat Rabbits
Source: Animals (Basel). 2026 Jun 11;16(12):1807. doi: 10.3390/ani16121807 (PMC13295800; doi:10.3390/ani16121807)
Supplement: Supplementary file 1 [file animals-16-01807-s001.zip › animals-4349403-Figure S1-Representative image showing morphology measurement in intestinal tissue. Crypt depth (yellow arrows), Villus length (red arrows), and Mucosal th.pdf]

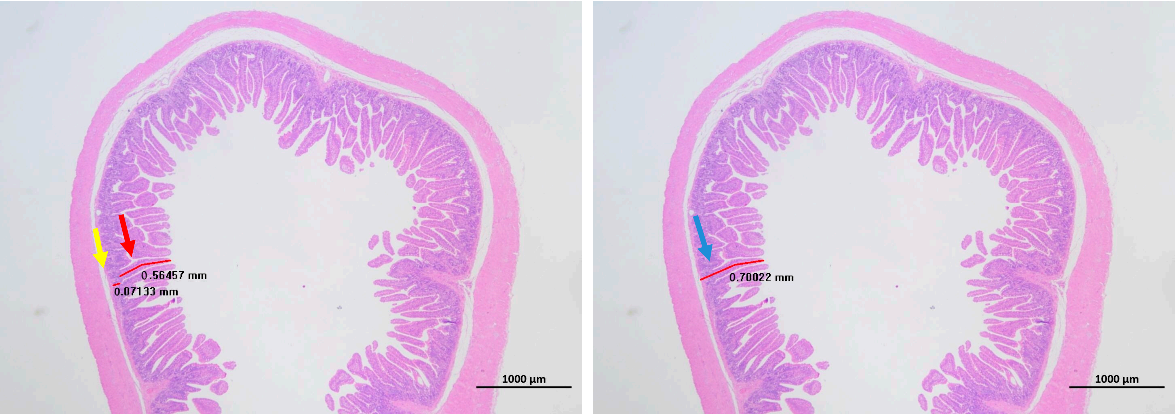

Figure S1. Representative image showing morphology measurement in intestinal tissue. Crypt depth (yellow arrows), Villus length (red arrows), and Mucosal thickness (blue arrows) are indicated
