# Supplementary material for: Dietary Coenzyme Q10 Supplementation Enhances Meat Quality, Nutritional Profile, and Antioxidant Status in Meat Rabbits
Source: Animals (Basel). 2026 Jun 11;16(12):1807. doi: 10.3390/ani16121807 (PMC13295800; doi:10.3390/ani16121807)
Supplement: Supplementary file 1 [file animals-16-01807-s001.zip › animals-4349403-Table S1. Composition and nutrient levels of the commercial pellet diet (air-dry basis, %). .pdf]

Table S1. Composition and nutrient levels of the commercial pellet diet (air-dry basis, %)

Ingredient	Content	Nutrient levels[2]	Content
Corn	25	Digestible energy (MJ/kg)	9.73
Soybean meal	7	Crude protein (%)	14.62
Peanut meal	6	Ether extract (%)	2.97
Bran	18	Crude fibre (%)	14.33
Alfalfa meal	25	Crude ash (%)	8.93
Peanut seedling powder	14	Calcium (%)	0.99
$CaHPO_4$	0.6	Phosphorus (%)	0.62
Salt	0.4		
Premix[1]	4.0		
Total/%	100		

[1] The premix provided the following per kg of diets: VA 120000 IU, VB1 30 mg, VB2 200 mg, VD3 20000 IU, VE 350 IU, VK3 30 mg, Fe (as ferrous sulfate) 1000 mg, Cu (as cupric sulfate) 440 mg, Zn (as zinc sulfate) 1035 mg, Mn (as manganese sulfate) 477 mg, NaCl 5%~6%.

[2] The digestible energy of the nutritional level was calculated, while the others were measured.
